# Supplementary material for: Construction of à la carte QconCAT protein standards for multiplexed quantification of user-specified target proteins
Source: BMC Biol. 2021 Sep 8;19:195. doi: 10.1186/s12915-021-01135-9 (PMC8425055; doi:10.1186/s12915-021-01135-9)
Supplement: Supplementary file 2 — Additional file 2: Table 1. Proteins/peptides used in ALACAT B. Figure S1. Extracted ion chromatogram and peptide coverage map for short ALACAT 301. Figure S2. Extracted ion chromatogram and peptide coverage map for short ALACAT 302. Figure S3. Extracted ion chromatogram and peptide coverage map for short ALACAT 301. Figure S4. Extracted ion chromatogram and peptide coverage map for short ALACAT 304. Figure S5. Extracted ion chromatogram and peptide coverage map for short ALACAT 305. Figure S6. Extracted ion chromatogram and peptide coverage map for long ALACAT B. Figure S7. Wheat germ proteins present in purified QconCATs. Figure S8. Uncropped SDS-PAGE images. [file 12915_2021_1135_MOESM2_ESM.pptx]

## Slide 1
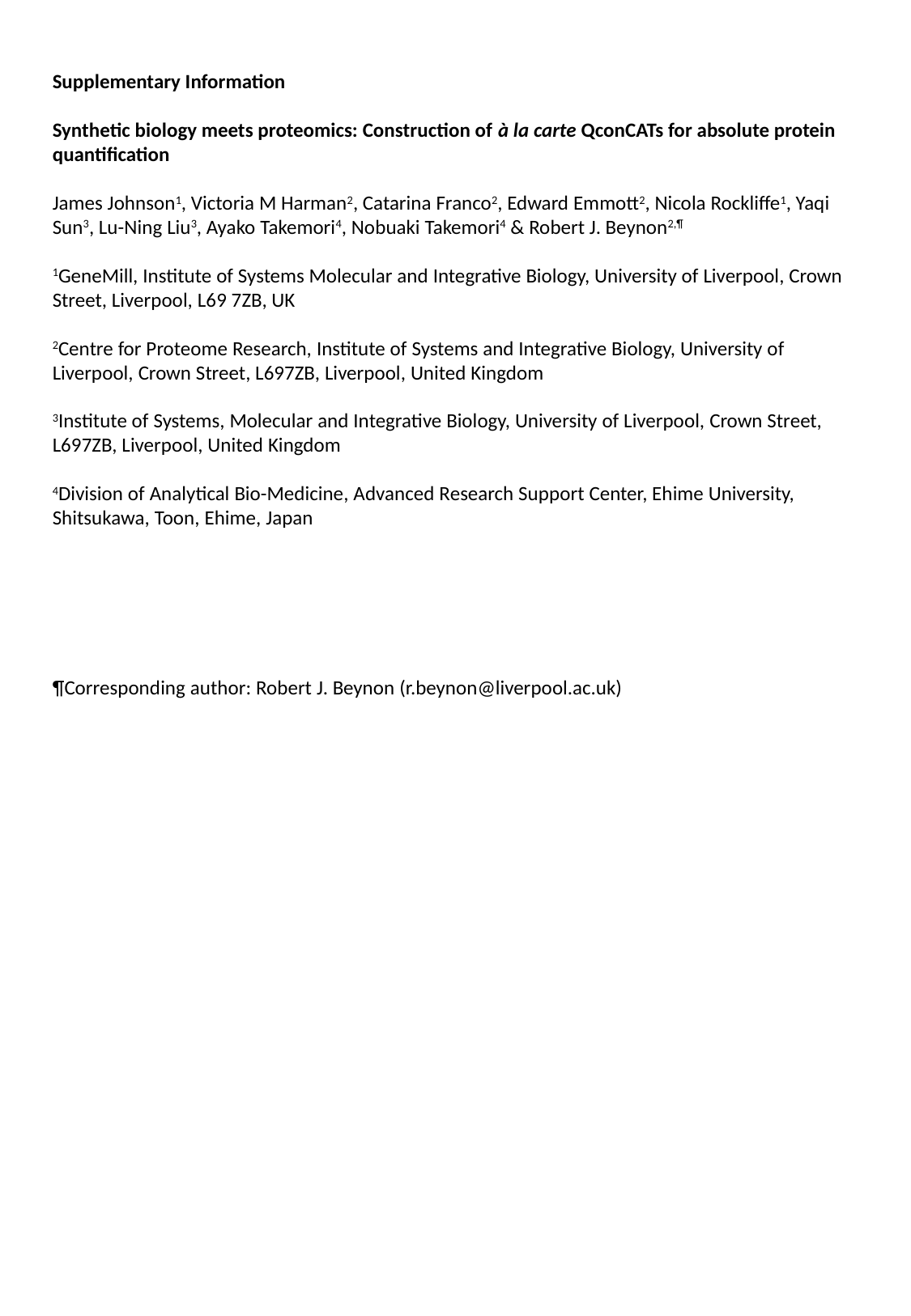

Supplementary Information
Synthetic biology meets proteomics: Construction of à la carte QconCATs for absolute protein quantification
James Johnson1, Victoria M Harman2, Catarina Franco2, Edward Emmott2, Nicola Rockliffe1, Yaqi Sun3, Lu-Ning Liu3, Ayako Takemori4, Nobuaki Takemori4 & Robert J. Beynon2,¶
1GeneMill, Institute of Systems Molecular and Integrative Biology, University of Liverpool, Crown Street, Liverpool, L69 7ZB, UK
2Centre for Proteome Research, Institute of Systems and Integrative Biology, University of Liverpool, Crown Street, L697ZB, Liverpool, United Kingdom
3Institute of Systems, Molecular and Integrative Biology, University of Liverpool, Crown Street, L697ZB, Liverpool, United Kingdom
4Division of Analytical Bio-Medicine, Advanced Research Support Center, Ehime University, Shitsukawa, Toon, Ehime, Japan
¶Corresponding author: Robert J. Beynon (r.beynon@liverpool.ac.uk)

## Slide 2
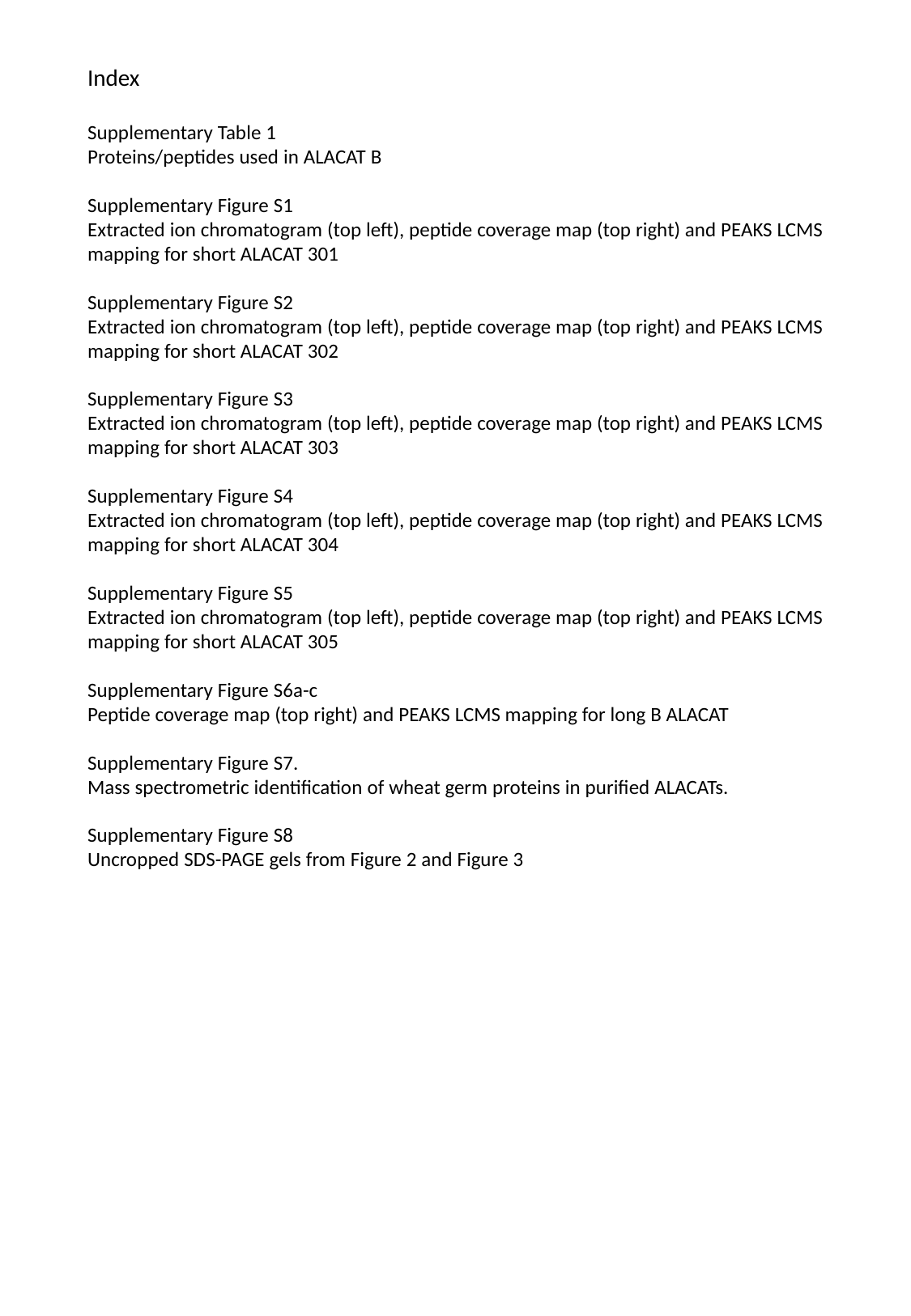

Index
Supplementary Table 1
Proteins/peptides used in ALACAT B
Supplementary Figure S1
Extracted ion chromatogram (top left), peptide coverage map (top right) and PEAKS LCMS mapping for short ALACAT 301
Supplementary Figure S2
Extracted ion chromatogram (top left), peptide coverage map (top right) and PEAKS LCMS mapping for short ALACAT 302
Supplementary Figure S3
Extracted ion chromatogram (top left), peptide coverage map (top right) and PEAKS LCMS mapping for short ALACAT 303
Supplementary Figure S4
Extracted ion chromatogram (top left), peptide coverage map (top right) and PEAKS LCMS mapping for short ALACAT 304
Supplementary Figure S5
Extracted ion chromatogram (top left), peptide coverage map (top right) and PEAKS LCMS mapping for short ALACAT 305
Supplementary Figure S6a-c
Peptide coverage map (top right) and PEAKS LCMS mapping for long B ALACAT
Supplementary Figure S7.
Mass spectrometric identification of wheat germ proteins in purified ALACATs.
Supplementary Figure S8
Uncropped SDS-PAGE gels from Figure 2 and Figure 3

## Slide 3
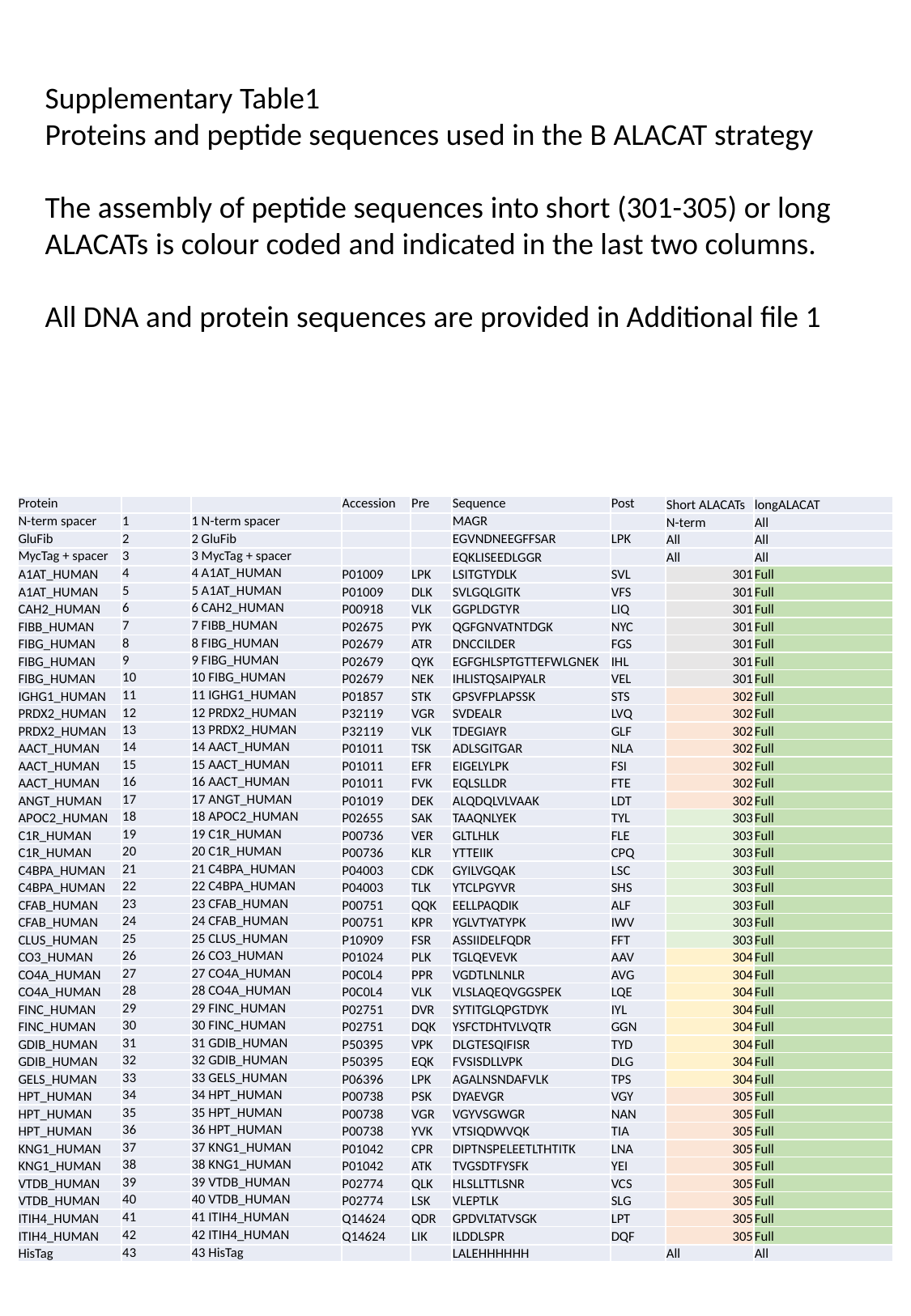

Supplementary Table1
Proteins and peptide sequences used in the B ALACAT strategy
The assembly of peptide sequences into short (301-305) or long ALACATs is colour coded and indicated in the last two columns.
All DNA and protein sequences are provided in Additional file 1
| Protein | | | Accession | Pre | Sequence | Post | Short ALACATs | longALACAT |
| --- | --- | --- | --- | --- | --- | --- | --- | --- |
| N-term spacer | 1 | 1 N-term spacer | | | MAGR | | N-term | All |
| GluFib | 2 | 2 GluFib | | | EGVNDNEEGFFSAR | LPK | All | All |
| MycTag + spacer | 3 | 3 MycTag + spacer | | | EQKLISEEDLGGR | | All | All |
| A1AT\_HUMAN | 4 | 4 A1AT\_HUMAN | P01009 | LPK | LSITGTYDLK | SVL | 301 | Full |
| A1AT\_HUMAN | 5 | 5 A1AT\_HUMAN | P01009 | DLK | SVLGQLGITK | VFS | 301 | Full |
| CAH2\_HUMAN | 6 | 6 CAH2\_HUMAN | P00918 | VLK | GGPLDGTYR | LIQ | 301 | Full |
| FIBB\_HUMAN | 7 | 7 FIBB\_HUMAN | P02675 | PYK | QGFGNVATNTDGK | NYC | 301 | Full |
| FIBG\_HUMAN | 8 | 8 FIBG\_HUMAN | P02679 | ATR | DNCCILDER | FGS | 301 | Full |
| FIBG\_HUMAN | 9 | 9 FIBG\_HUMAN | P02679 | QYK | EGFGHLSPTGTTEFWLGNEK | IHL | 301 | Full |
| FIBG\_HUMAN | 10 | 10 FIBG\_HUMAN | P02679 | NEK | IHLISTQSAIPYALR | VEL | 301 | Full |
| IGHG1\_HUMAN | 11 | 11 IGHG1\_HUMAN | P01857 | STK | GPSVFPLAPSSK | STS | 302 | Full |
| PRDX2\_HUMAN | 12 | 12 PRDX2\_HUMAN | P32119 | VGR | SVDEALR | LVQ | 302 | Full |
| PRDX2\_HUMAN | 13 | 13 PRDX2\_HUMAN | P32119 | VLK | TDEGIAYR | GLF | 302 | Full |
| AACT\_HUMAN | 14 | 14 AACT\_HUMAN | P01011 | TSK | ADLSGITGAR | NLA | 302 | Full |
| AACT\_HUMAN | 15 | 15 AACT\_HUMAN | P01011 | EFR | EIGELYLPK | FSI | 302 | Full |
| AACT\_HUMAN | 16 | 16 AACT\_HUMAN | P01011 | FVK | EQLSLLDR | FTE | 302 | Full |
| ANGT\_HUMAN | 17 | 17 ANGT\_HUMAN | P01019 | DEK | ALQDQLVLVAAK | LDT | 302 | Full |
| APOC2\_HUMAN | 18 | 18 APOC2\_HUMAN | P02655 | SAK | TAAQNLYEK | TYL | 303 | Full |
| C1R\_HUMAN | 19 | 19 C1R\_HUMAN | P00736 | VER | GLTLHLK | FLE | 303 | Full |
| C1R\_HUMAN | 20 | 20 C1R\_HUMAN | P00736 | KLR | YTTEIIK | CPQ | 303 | Full |
| C4BPA\_HUMAN | 21 | 21 C4BPA\_HUMAN | P04003 | CDK | GYILVGQAK | LSC | 303 | Full |
| C4BPA\_HUMAN | 22 | 22 C4BPA\_HUMAN | P04003 | TLK | YTCLPGYVR | SHS | 303 | Full |
| CFAB\_HUMAN | 23 | 23 CFAB\_HUMAN | P00751 | QQK | EELLPAQDIK | ALF | 303 | Full |
| CFAB\_HUMAN | 24 | 24 CFAB\_HUMAN | P00751 | KPR | YGLVTYATYPK | IWV | 303 | Full |
| CLUS\_HUMAN | 25 | 25 CLUS\_HUMAN | P10909 | FSR | ASSIIDELFQDR | FFT | 303 | Full |
| CO3\_HUMAN | 26 | 26 CO3\_HUMAN | P01024 | PLK | TGLQEVEVK | AAV | 304 | Full |
| CO4A\_HUMAN | 27 | 27 CO4A\_HUMAN | P0C0L4 | PPR | VGDTLNLNLR | AVG | 304 | Full |
| CO4A\_HUMAN | 28 | 28 CO4A\_HUMAN | P0C0L4 | VLK | VLSLAQEQVGGSPEK | LQE | 304 | Full |
| FINC\_HUMAN | 29 | 29 FINC\_HUMAN | P02751 | DVR | SYTITGLQPGTDYK | IYL | 304 | Full |
| FINC\_HUMAN | 30 | 30 FINC\_HUMAN | P02751 | DQK | YSFCTDHTVLVQTR | GGN | 304 | Full |
| GDIB\_HUMAN | 31 | 31 GDIB\_HUMAN | P50395 | VPK | DLGTESQIFISR | TYD | 304 | Full |
| GDIB\_HUMAN | 32 | 32 GDIB\_HUMAN | P50395 | EQK | FVSISDLLVPK | DLG | 304 | Full |
| GELS\_HUMAN | 33 | 33 GELS\_HUMAN | P06396 | LPK | AGALNSNDAFVLK | TPS | 304 | Full |
| HPT\_HUMAN | 34 | 34 HPT\_HUMAN | P00738 | PSK | DYAEVGR | VGY | 305 | Full |
| HPT\_HUMAN | 35 | 35 HPT\_HUMAN | P00738 | VGR | VGYVSGWGR | NAN | 305 | Full |
| HPT\_HUMAN | 36 | 36 HPT\_HUMAN | P00738 | YVK | VTSIQDWVQK | TIA | 305 | Full |
| KNG1\_HUMAN | 37 | 37 KNG1\_HUMAN | P01042 | CPR | DIPTNSPELEETLTHTITK | LNA | 305 | Full |
| KNG1\_HUMAN | 38 | 38 KNG1\_HUMAN | P01042 | ATK | TVGSDTFYSFK | YEI | 305 | Full |
| VTDB\_HUMAN | 39 | 39 VTDB\_HUMAN | P02774 | QLK | HLSLLTTLSNR | VCS | 305 | Full |
| VTDB\_HUMAN | 40 | 40 VTDB\_HUMAN | P02774 | LSK | VLEPTLK | SLG | 305 | Full |
| ITIH4\_HUMAN | 41 | 41 ITIH4\_HUMAN | Q14624 | QDR | GPDVLTATVSGK | LPT | 305 | Full |
| ITIH4\_HUMAN | 42 | 42 ITIH4\_HUMAN | Q14624 | LIK | ILDDLSPR | DQF | 305 | Full |
| HisTag | 43 | 43 HisTag | | | LALEHHHHHH | | All | All |

## Slide 4
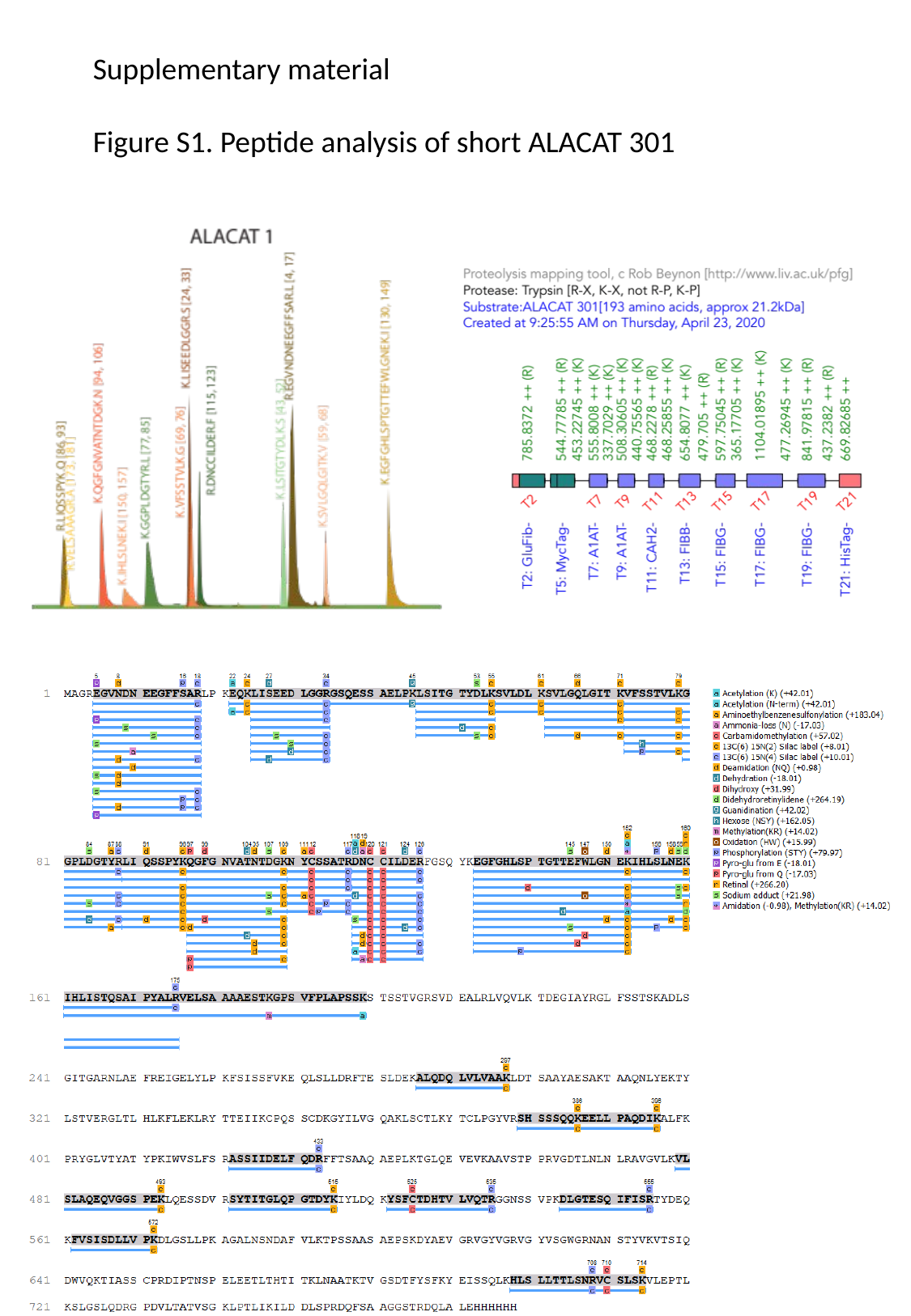

Supplementary material
Figure S1. Peptide analysis of short ALACAT 301

## Slide 5
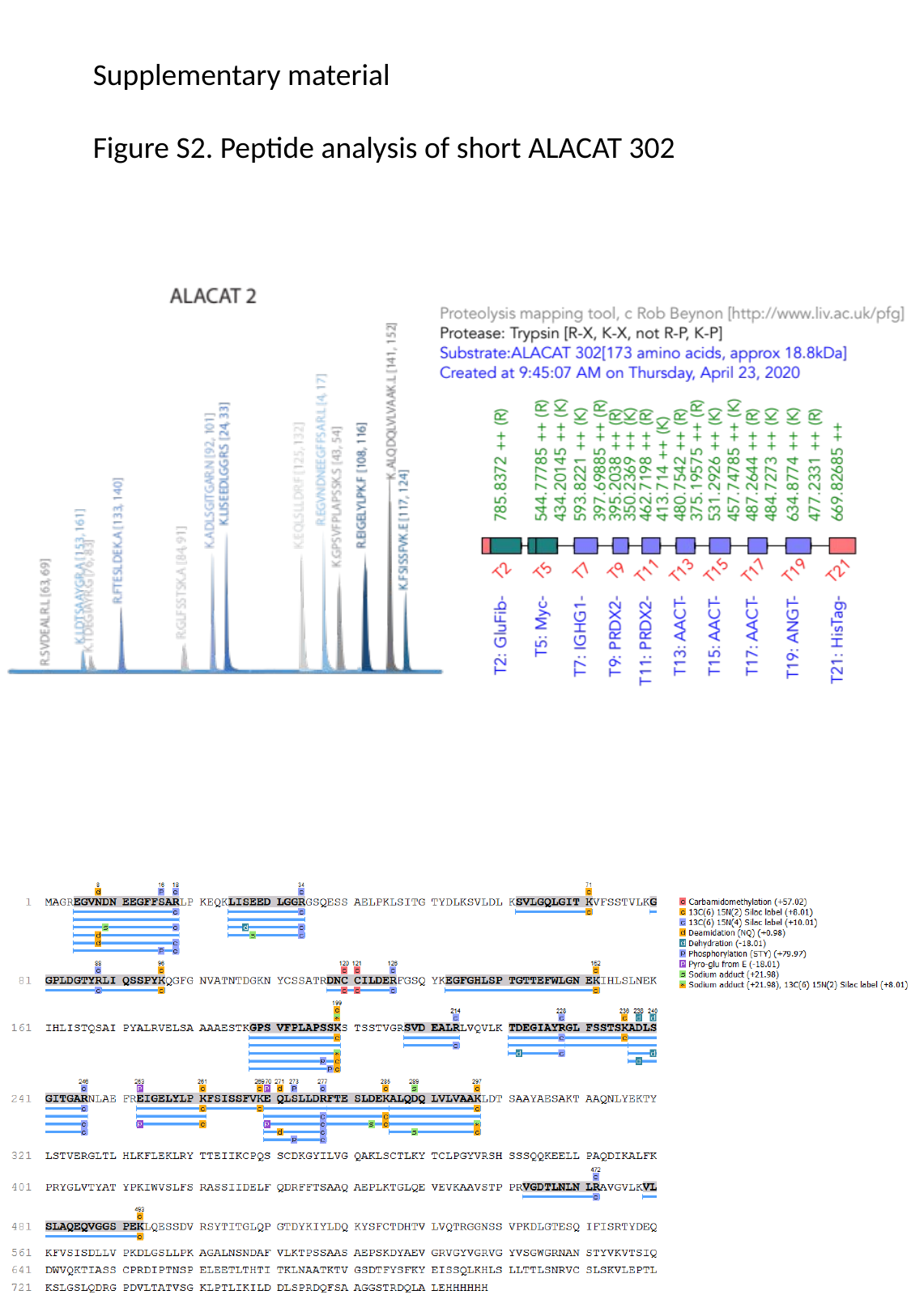

Supplementary material
Figure S2. Peptide analysis of short ALACAT 302

## Slide 6
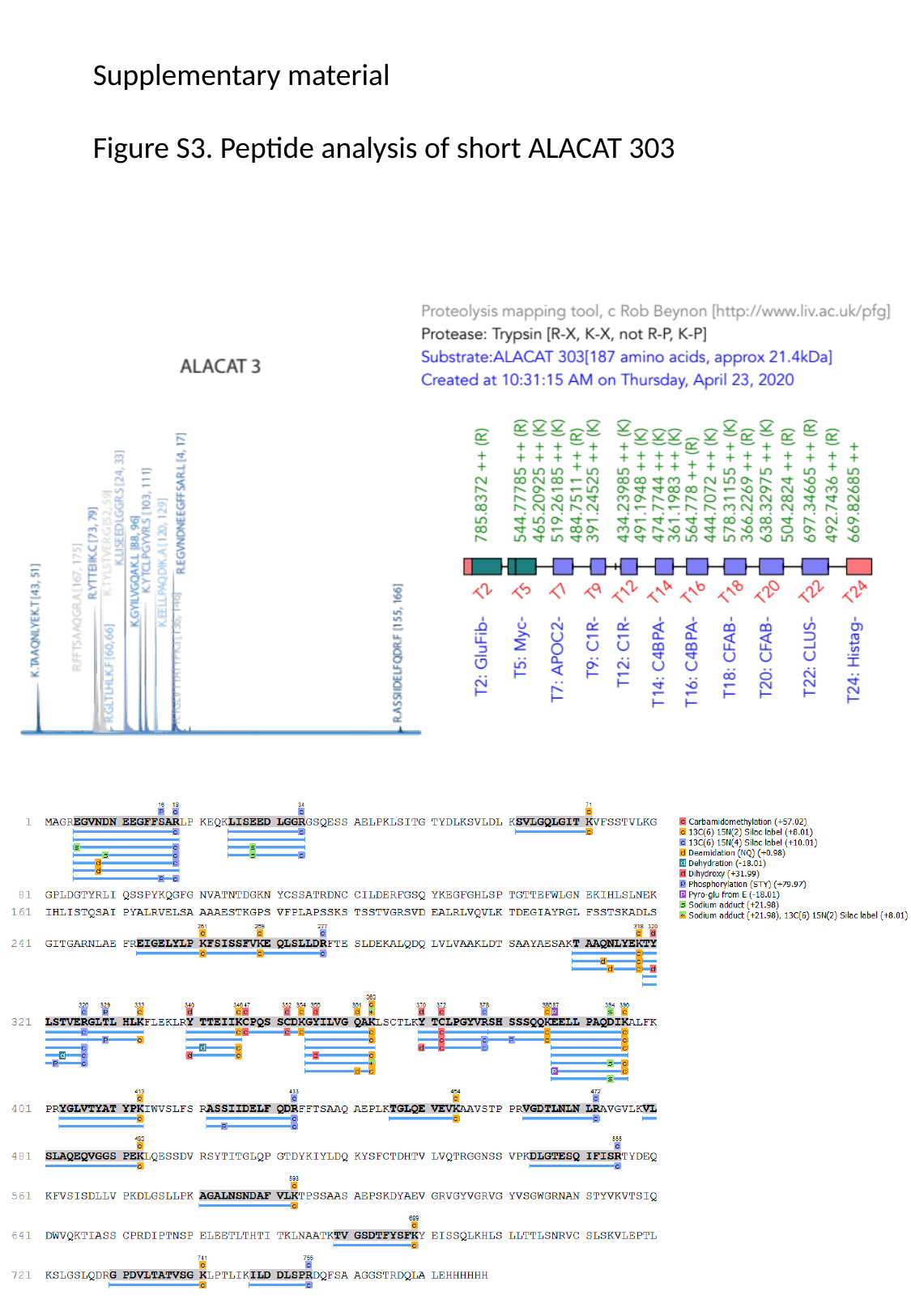

Supplementary material
Figure S3. Peptide analysis of short ALACAT 303

## Slide 7
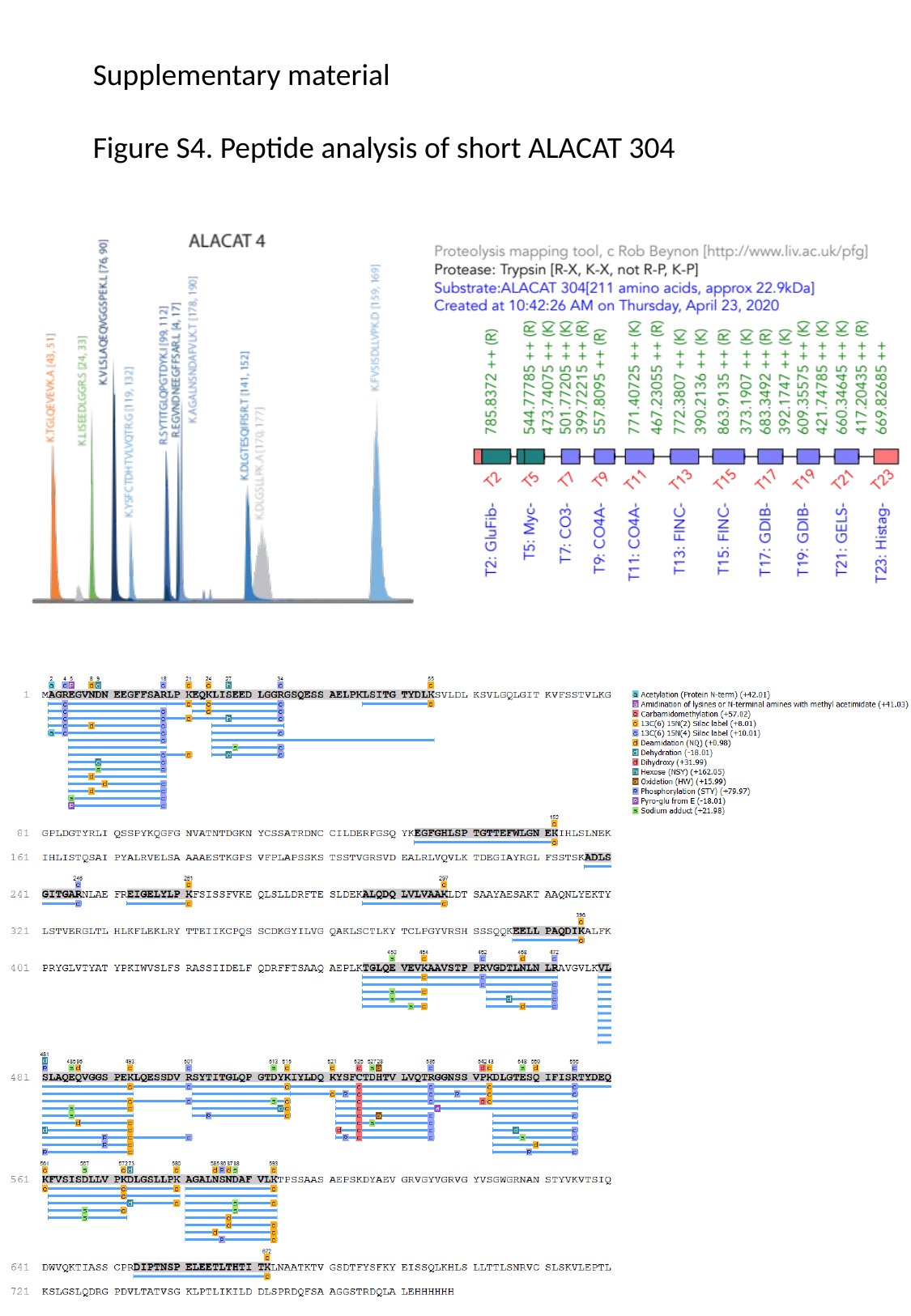

Supplementary material
Figure S4. Peptide analysis of short ALACAT 304

## Slide 8
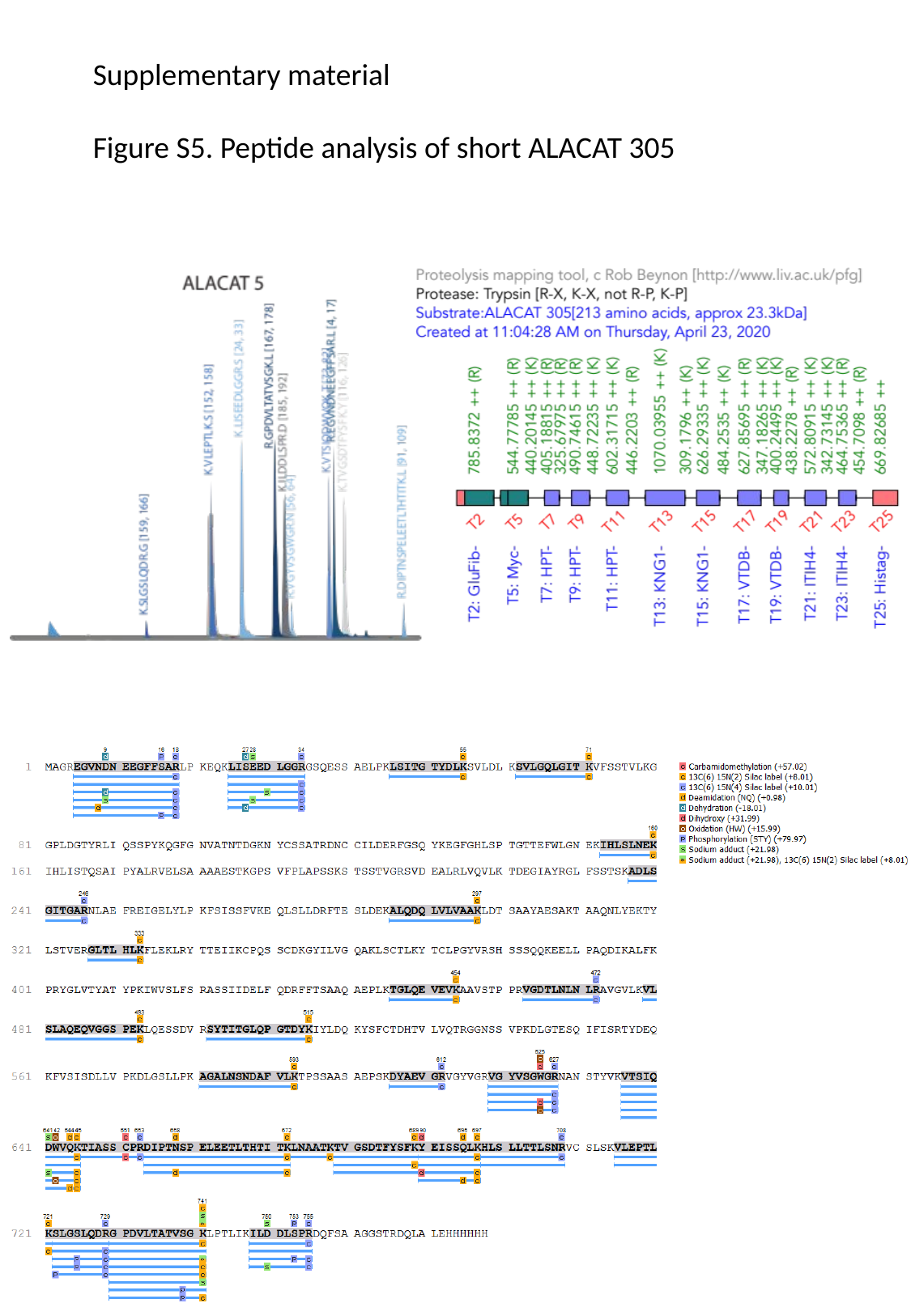

Supplementary material
Figure S5. Peptide analysis of short ALACAT 305

## Slide 9
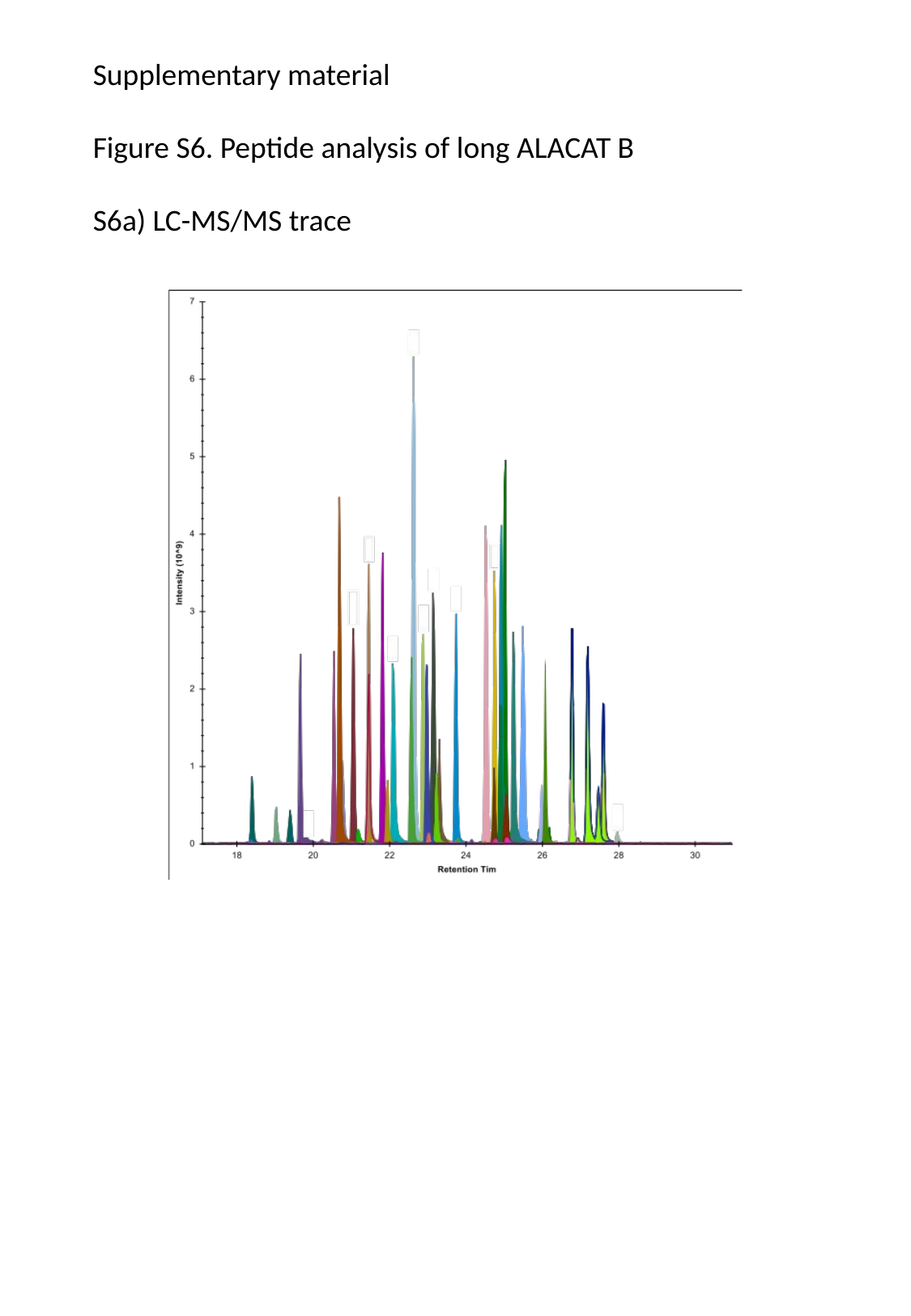

Supplementary material
Figure S6. Peptide analysis of long ALACAT B
S6a) LC-MS/MS trace

## Slide 10
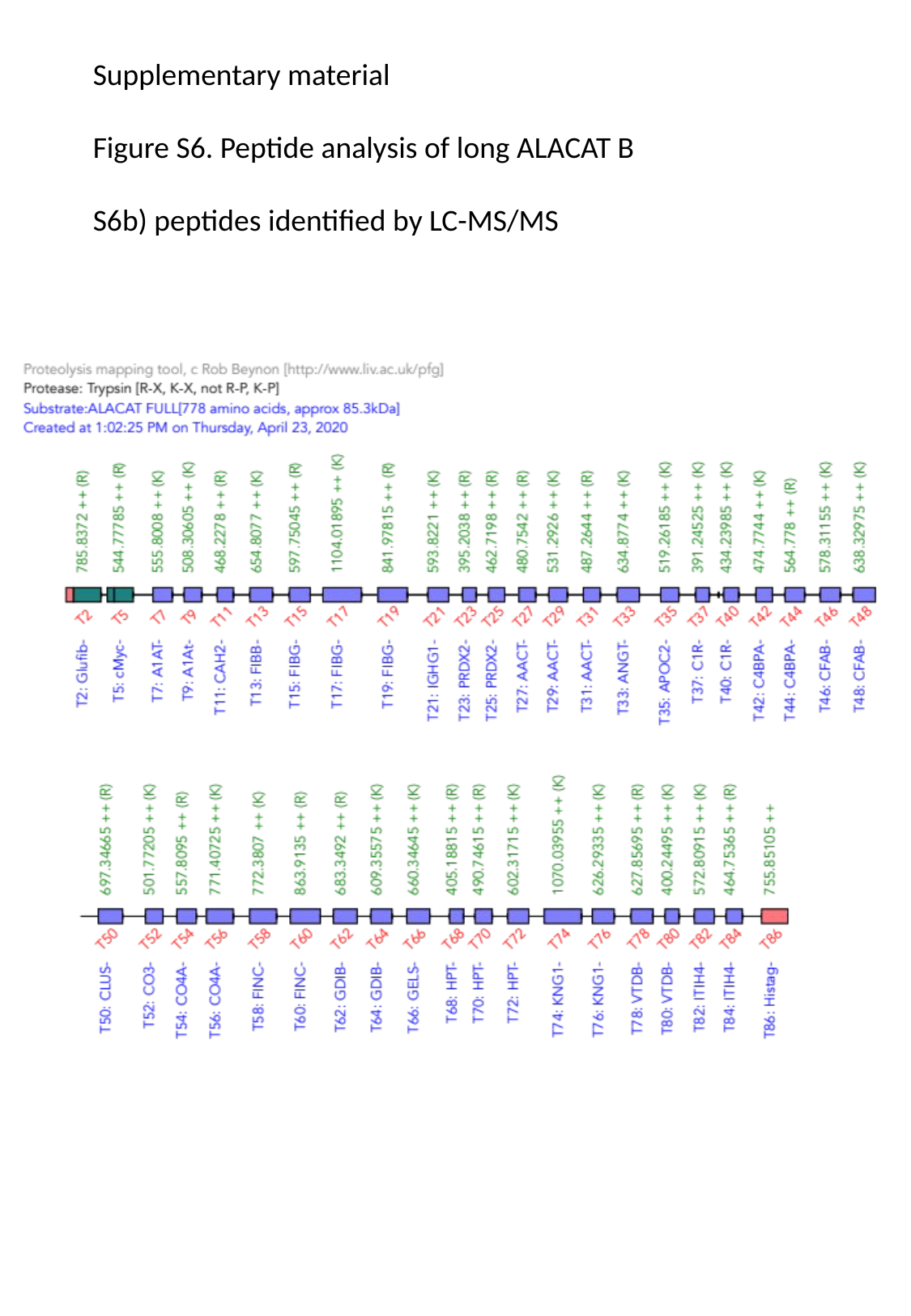

Supplementary material
Figure S6. Peptide analysis of long ALACAT B
S6b) peptides identified by LC-MS/MS

## Slide 11
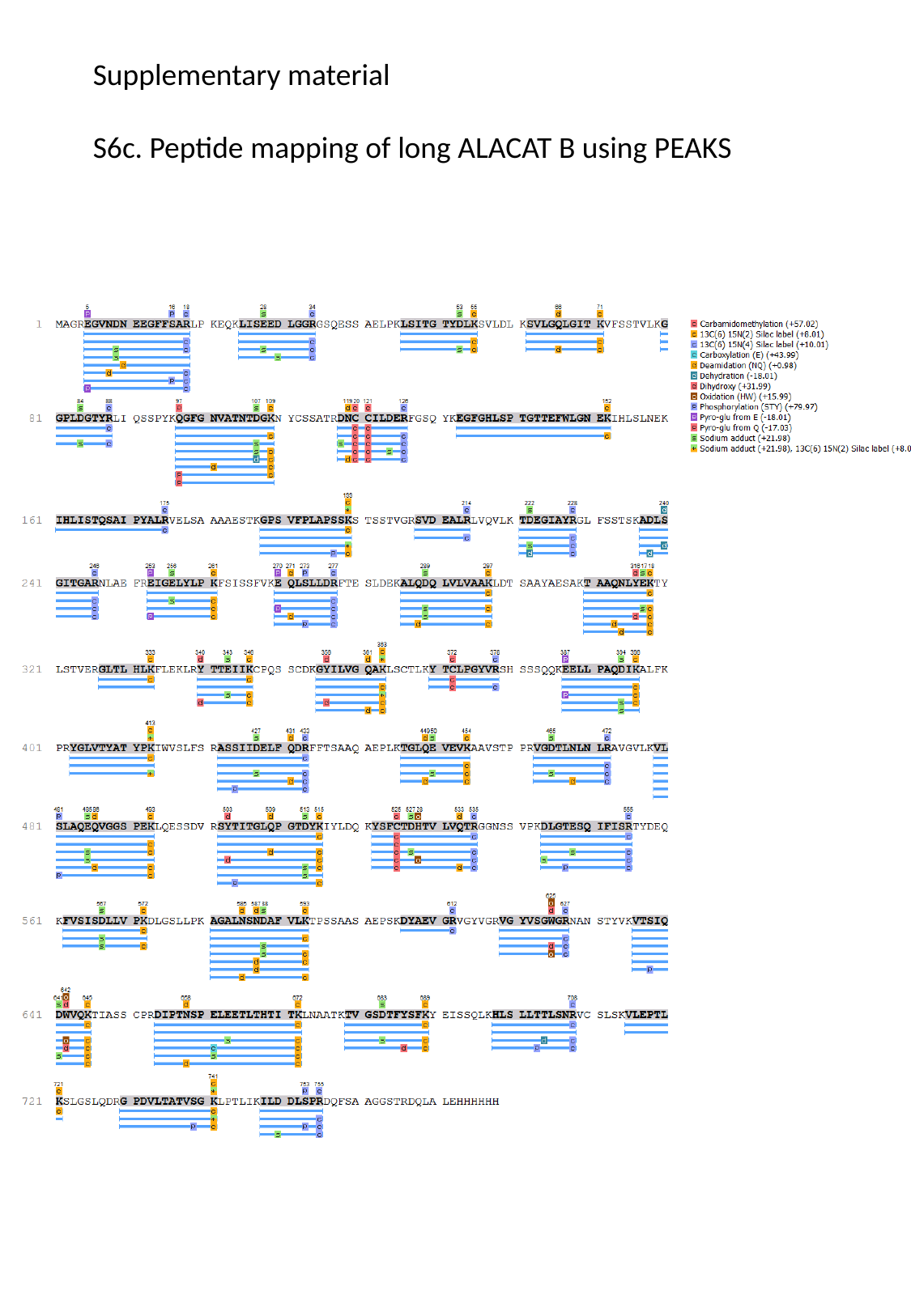

Supplementary material
S6c. Peptide mapping of long ALACAT B using PEAKS

## Slide 12
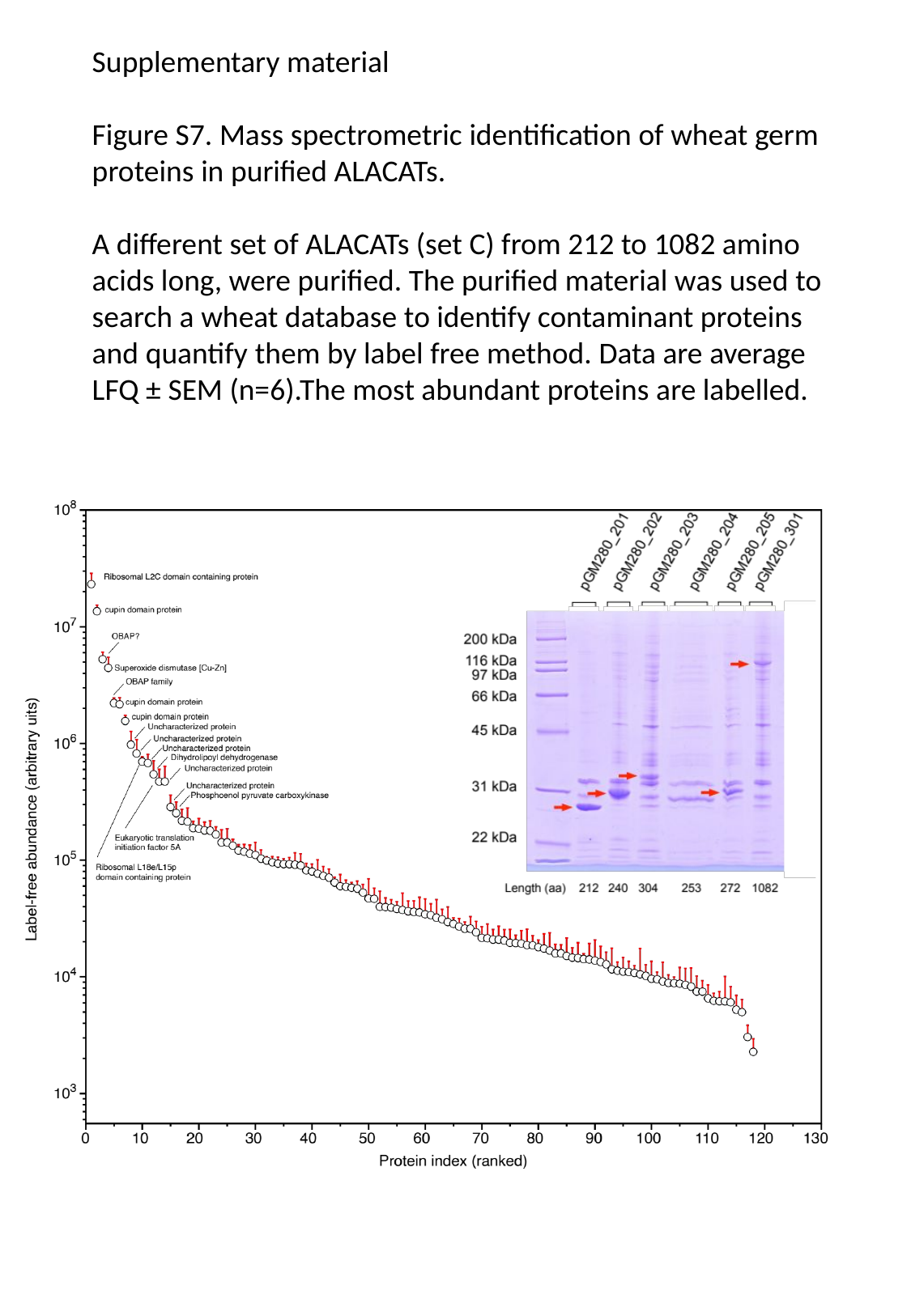

Supplementary material
Figure S7. Mass spectrometric identification of wheat germ proteins in purified ALACATs.
A different set of ALACATs (set C) from 212 to 1082 amino acids long, were purified. The purified material was used to search a wheat database to identify contaminant proteins and quantify them by label free method. Data are average LFQ ± SEM (n=6).The most abundant proteins are labelled.

## Slide 13
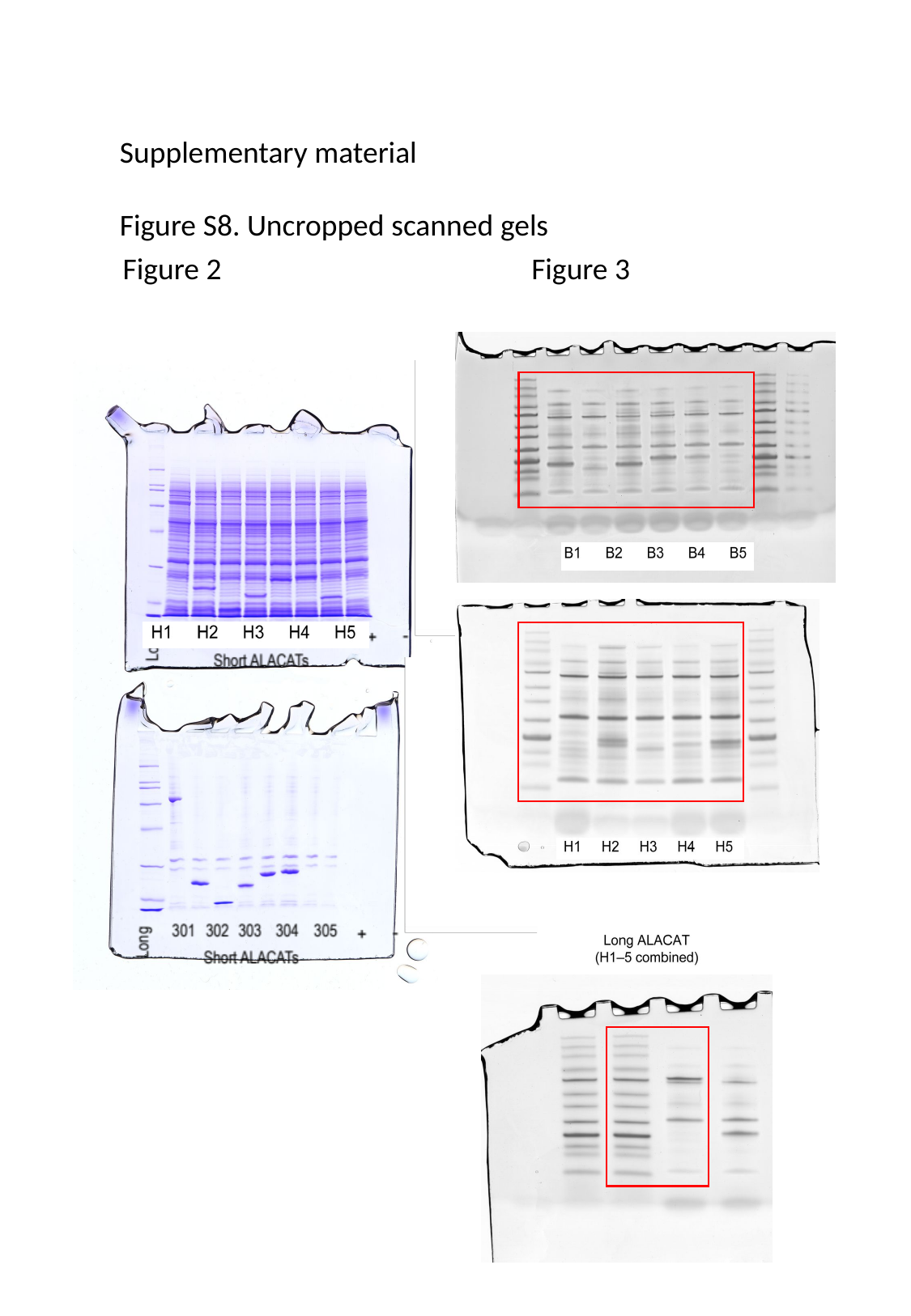

Supplementary material
Figure S8. Uncropped scanned gels
Figure 2
Figure 3
